# Clinical Practice Associated with a Switch from and to Ziprasidone during Routine Inpatient Treatment of Patients with Schizophrenia

**DOI:** 10.1155/2011/317368

**Published:** 2011-10-27

**Authors:** Matthias J. Müller

**Affiliations:** Clinic for Psychiatry and Psychotherapy Giessen and Marburg, Vitos Clinical Centre Giessen-Marburg, Academic Teaching Hospital, University of Giessen, Licher Strasse 106, 35392 Giessen, Germany

## Abstract

Ziprasidone (ZIP) shows a low propensity for metabolic side effects but can prolong QTc time. It is unclear how these features translate into clinical reality. Charts of inpatients with schizophrenia and switched from (ZIP − , *n* = 27) or to ZIP (ZIP + , *n* = 24) were reviewed. Clinical data including documented switch reasons were anonymously analyzed. Comorbidity, body mass index (BMI) at admission, illness severity, side effects, illness duration, and length of stay were comparable in both groups. About 2/3 of ZIP+ were women (1/3 of ZIP − , *P* = 0.035); ZIP+ patients were younger (*P* = 0.017), had higher BMI values (*P* = 0.042), and received higher chlorpromazine equivalents before switch (*P* = 0.004) whereas ZIP doses were comparable (136 versus 141 mg/d). More patients in ZIP− versus ZIP+ were switched because of previous weight gain (*P* = 0.006) and depression (*P* = 0.085) whereas single reasons for ZIP− versus ZIP+ were mainly persisting positive symptoms (*P* = 0.089) and patients' choice (*P* = 0.10). The results of the naturalistic study corroborate controlled trials.

## 1. Introduction

Ziprasidone (ZIP) is one of the second-generation antipsychotics (SGA) with proven low propensity for unwanted metabolic changes (weight gain, hyperlipidemia, hyperglycemia, etc.) [1, 2]. Thus, together with aripiprazole, ZIP is an alternative to conventional, first-generation antipsychotics (FGA), particularly if metabolic syndrome has to be avoided or if metabolic changes have occurred under treatment with other SGAs or FGAs. 

ZIP was approved in 2001 as the fifth SGA by the FDA for treatment of schizophrenia (approval in Germany 2002). The approval has been extended for acute treatment of mania and mixed states associated with bipolar disorder. ZIP has a highly selective affinity for 5-HT2A receptors relative to D2 and 5-HT2C receptors compared to other FGAs and SGAs and shows also high blocking affinity for alpha-adrenergic receptors and a moderate antagonistic affinity for histamine H1-receptors [3]. This pharmacodynamic profile of ZIP explains the antipsychotic effects and some of the side effects of ZIP such as sedation and orthostasis [4, 5]. A modest inhibition of synaptic serotonin and norepinephrine reuptake of ZIP has been suggested to be involved in some antidepressant properties, although clinical significance is questionable [6]. ZIP is hepatically metabolized by aldehyde oxidase and via cytochrome P450 3A4 (CYP3A4). Absorption and sufficient bioavailability are dependent on sufficient preceding food intake (>500 kcal) [7, 8]. ZIP can slightly increase the QTc interval and the risk of potentially fatal arrhythmias [9]. 

In recent meta-analyses [2, 10] the effects of ZIP were compared with other SGAs in patients with schizophrenia and related disorders. According to these data, the rate of premature study discontinuation with ZIP was very high (59.1%) and early discontinuation—due to any reason—was higher than with olanzapine and risperidone, but not higher than with other SGAs. Additionally, ZIP was found to be slightly less efficacious than amisulpride, olanzapine, and risperidone in a meta-analysis [2]. Due to limited data no significant differences in tolerability between ZIP and amisulpride or clozapine were found, but ZIP produced consistently less weight gain than olanzapine, quetiapine, or risperidone. ZIP was associated with less cholesterol increase than olanzapine, quetiapine and risperidone. ZIP produced slightly higher rates of extrapyramidal side effects than olanzapine, but lower than risperidone. Prolactin increase with ZIP was greater than with quetiapine, but lower than with risperidone. In summary, despite a slightly lower efficacy compared to amisulpride, olanzapine, and risperidone, the greatest advantage of ZIP is a low propensity to induce weight gain [1, 2, 10, 11] and associated adverse effects (metabolic syndrome). 

Open studies reporting on the switch from other antipsychotics to ZIP have found beneficial effects of ZIP on metabolic changes and no worsening of psychopathology [5, 12–15]. These studies were partially extensions of randomized controlled studies or open studies designed to investigate clinical effects of ZIP. However, whereas these studies have reported effects of switching to ZIP (ZIP+) under more or less standardized conditions, little is known regarding the reasons for a switch from ZIP (ZIP−) to other antipsychotics and ZIP+ under real-life conditions. Therefore, the clinical database of a routine care psychiatric hospital was searched for patients with ZIP+ and ZIP− to explore the characteristics of both patients' groups and main reasons for switch.

## 2. Materials and Methods

We searched for all inpatients of the psychiatric state hospital (107 inpatient beds) in Marburg, Germany, hospitalized in the years 2007–2009 using the following criteria: diagnosis of schizophrenic disorder according to ICD-10 (F20), documented switch from other antipsychotics to ZIP (antipsychotic monotherapy; ZIP+), or documented switch from ZIP (antipsychotic monotherapy) to other antipsychotics (ZIP−) within the first two weeks of hospitalization.

The available guidelines for schizophrenia treatment, for rationale, individualized pharmacotherapy, and for informed consent are part of the quality management of the clinic and summarized in a clinical pathway. Nonetheless, switch of antipsychotic drugs is to a great extent still left up to the discretion of physicians and patients.

A total group of 1059 inpatients (1737 cases) with schizophrenia (ICD-10 F20) was found in the register. Out of this population, 27 patients (2.5%) were switched to ziprasidone (ZIP+), and 24 patients (2.3%) were switched from ziprasidone to other antipsychotics (ZIP−) during hospitalization and routine inpatient treatment and were eligible according to the above criteria. In total, eight psychiatrists and one attending were involved in the treatment of ZIP+ and ZIP− patients. 

A chart analysis of these two groups was carried out by two independent experienced clinicians not involved in the treatment of the patients. Severity of global illness at admission and discharge were judged with 7-point global assessment ratings (clinical global impression, CGI; (1) normal, not at all ill; (2) borderline mentally ill; (3) mildly ill; (4) moderately ill; (5) markedly ill; (6) severely ill; (7) extremely ill) using all available information. Accordingly, global severity of side effects was judged on a pragmatic 4-point scale (0 = none; 1 = mild; 2 = moderate; 3 = severe). The severity rating of side effects comprised all documented aspects of possibly drug-related signs and symptoms, for example, extrapyramidal symptoms (EPS), sedation, agitation, and sexual dysfunction. Variation in reporting and documentation made it impossible to analyze specific side effects.

Only patients with documented switch were included in the present study. All documented reasons for ZIP+ or ZIP− were listed and categorized in two steps. The first categorization step comprised nine switch reasons (multiple entries were possible): persisting positive symptoms, severe and burdensome depression, severe and disabling negative symptoms, interfering sedation, intolerable weight gain, severe agitation, intolerable EPS, other side effects, and the patient's choice. The latter consisted of admitted discontinuation and noncompliance and of alleged specific desires to change the antipsychotic without further explanation. The second step of categorizing reasons led to three broad categories of one main switch reason per patient: lack of effectiveness, side effects, and patients' choice. As more than one reason for a switch was possible at the first categorization step, the assumed single main reason (second step) was finally judged using all available information. 

Body weight and body mass indices were obtained from the charts at admission or within 1 week after admission. Further laboratory and metabolic parameters and changes could not be analyzed. 

Psychotropic medication at discharge and antipsychotic medication directly before switching was assessed in both groups. Chlorpromazine equivalents (CPZE) were calculated on the basis of daily doses for antipsychotic medication according to the literature [16–18]. Clinical data were compared between both groups (ZIP−/ZIP+). For categorical data, *χ* ^2^ tests were used, for continuous data *t*-tests for independent groups were applied. The level of statistical significance was set at *α* = 0.05; results with *P* < 0.10 were reported as statistical trend due to the exploratory nature of the study.

## 3. Results

In both groups switch of antipsychotics occurred in most cases at admission or by cross-tapering within 10 days (Table 1) due to acute exacerbation requiring hospitalization. Table 1 shows clinical data of the two groups with patients switched either from or to ZIP. 

Significantly more female patients were switched to ZIP (*P* < 0.05); additionally, these patients were younger (*P* < 0.05) and had higher BMI values (*P* < 0.05) than patients switched from ZIP to other antipsychotics (ZIP+). At admission chlorpromazine equivalents (CPZE) were significantly higher in ZIP+, and after switching to ZIP+, CPZE were significantly lower at discharge (*P* < 0.01). ZIP daily doses were comparable in both groups (after or before switching, resp.). Comparably high proportions of patients in ZIP+ and ZIP− received psychotropic comedication (ZIP+ 79% versus ZIP− 82%). Clinical severity and side-effect judgements were comparable in both groups.  Table 2 shows the psychotropic medication before and after switching from or to ZIP.

The patterns of medication were similar in both groups, in the ZIP+ seven patients were switched from FGA to ZIP; in the ZIP+ group seven patients versus four in the ZIP− group received concomitant antidepressant treatment, one patient in ZIP+ received two antidepressants (sertraline + mirtazapine). In Table 3 clinical reasons for switching to or from ZIP are reported; in some cases more than one reason for switching was recorded. 

Significantly more patients were switched to ZIP because of weight gain during pretreatment (*P* < 0.05), depression, and extrapyramidal symptoms were found as reasons for a switch with a statistical tendency in favor of ZIP+ (*P* < 0.10). Reasons for a switch from ZIP were persisting positive symptoms and patients' choice with a slightly higher frequency (*P* ≤ 0.10) in ZIP− compared to ZIP+. 

**Figure 1:**
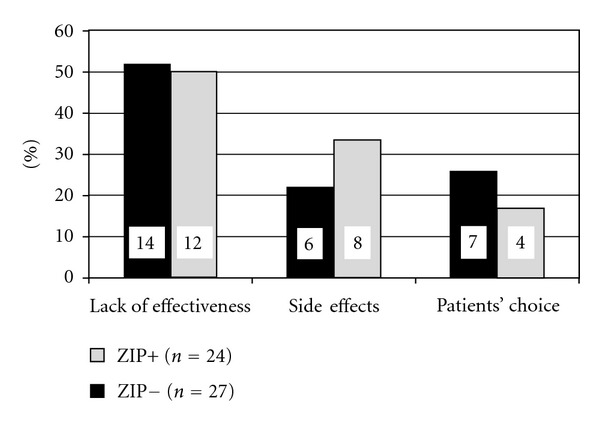
Main reasons for a switch from or to ziprasidone ZIP−, switch from ziprasidone; ZIP+, switch to ziprasidone.

The categorized main reasons for switch (lack of effectiveness, side effects, and patients' choice; only one per patient) were not significantly different between ZIP+ and ZIP− (*χ* ^2^ test, *P* = 0.39).  Figure 1 illustrates the distribution of categories in both groups.

The main reason for a switch from ZIP (ZIP−) was lack of effectiveness regarding positive, negative, or affective symptoms of schizophrenia (approximately 50%). Side effects and the subjective choice of patients possibly relating to both psychopathology and unwanted effects of treatment were nearly equally distributed (both approximately 25%).

## 4. Discussion

The switch to or from ZIP during routine clinical treatment of schizophrenia was reviewed in a German psychiatric state hospital. Data were retrospectively analyzed to investigate whether the properties of ZIP found in controlled studies and meta-analyses are implicitly or explicitly considered in real-life situations. A proportion of patients <10% received ZIP indicating that ZIP is not a first-line treatment for schizophrenia in Germany. If the low propensity of ZIP for unwanted metabolic changes compared to other antipsychotic compounds is clinically relevant, reasons for a switch to ZIP (ZIP+) should reflect this aspect compared to ZIP−. The results of the present study corroborate this hypothesis as more patients with ZIP+ were switched because of previous weight gain than patients with ZIP−. Moreover, BMI values at baseline were significantly higher at least in women with schizophrenia switched to ZIP (ZIP+) in comparison to ZIP−.

Despite guidelines and clinical pathways the decision to change an antipsychotic drug and which substance is used remains often on a consensus between the patient and the physicians who are in charge. Thus, the results of the present analysis are not independent of subjective aspects of clinical decision making [19]. According to available guidelines and recommendations, ZIP and aripiprazole are considered superior in patients with schizophrenia and weight or metabolic problems [19, 20]. However, in large-scale analyses of US data the physicians' adherence to these recommendations seems to be low [21]. In larger trials [2, 22] ZIP treatment of schizophrenia was discontinued in a high proportion of >50% of patients within 12–18 months, probably more than under other SGAs. This question could not be evaluated with the present data, as only a single switch (from or to ZIP) was focused in the present study, the switch occurred in the first two weeks of treatment, and patients were not followedup. Nonetheless, the patients' choice to change ZIP was one reason among others to switch in 1/3 and the main reason to switch from ZIP in 1/4 of patients in the present study and may reflect the early discontinuation found in meta-analyses which was not caused by metabolic effects or weight gain but by a lack of efficacy [2, 10].

Patients switched to ZIP were younger and had tentatively more affective symptoms and psychiatric comorbidity, and more women were in the ZIP+ group whereas subtype of schizophrenia, severity and duration of illness, and global severity of side-effect were not different between ZIP+ and ZIP−. These findings are in line with previous reports [14, 15, 23, 24]. The mean daily doses of ZIP were approximately 140 mg in both groups according to recommended and widely used doses of ZIP [25, 26]. The results can thus not confirm findings that higher doses of ZIP are more effective in some patients with schizophrenia [27] although the proportion of patients switched from ZIP due to apparent ineffectiveness was rather high. Switching to ZIP resulted in lower daily chlorpromazine equivalents for patients. This finding is conforming with recent suggestions [28] but could also have resulted from still questionable equivalent doses [18].

The methodological limitations of a retrospective chart analysis based on semistandardized clinical data hamper the generalization of the present findings. First, the low proportion of patients switched to or from ZIP within three years seems to reflect the actual market share of ZIP in Germany for schizophrenia treatment but resulted in a rather low sample size. Selection bias due to specific implicit treatment strategies in this single-site study cannot be ruled out completely despite an implemented clinical pathway for the treatment of schizophrenia. Mult-center studies could avoid this source of bias in the future. The data concerning psychopathology and the decision to switch were derived from the original charts whereas medication at admission and discharge were taken from discharge letters. Both parts of information can be questioned regarding reliability. However, continuous reports during the course of treatment and widely standardized discharge letters support the applied method. 

Furthermore, one could criticize that subjective “weight gain” and not objectively assessed body weight, body mass index, or laboratory measures of metabolic changes were reported as in controlled studies published in the literature. However, beside the fact that reliable data on weight before and after switch to or from ZIP were not obtainable objective weight gain during a rather short time period is of questionable value. From the clinical standpoint, the patients' view [22] seems to be much more important for treatment success and adherence. A recent study in first-episode patients showed that patients' attitudes beside positive symptoms and sexual dysfunction were the strongest predictors of treatment success [29]. In this perspective our data may offer some “ecological validity” in addition to controlled, but more artificial conditions in many randomized trials. Furthermore, the aspect of “shared decision making” contains the patients' view explicitly and is in the present study also reflected in the finding, that a great number of patients (14/51, 27%) were switched mainly due to their own choice. This was by tendency more frequently the case for a switch from ZIP.

## 5. Conclusions

In summary, the data reflect some findings from large randomized trials regarding the use of ZIP in the treatment of schizophrenia in a routine inpatient care setting but show also the limitations and restrictions of transferring such results into real life. Switching antipsychotics should be considered early in the course of treatment if effectiveness is lacking [30], and a switch due to side effects, particularly weight gain and metabolic changes, can be cost-effective [31] in the long run. In this regard, ZIP seems to be a reasonable option in clinical routine for the treatment of schizophrenia. However, medication decisions should always be made collaboratively between the patient and his treating clinician.

## Figures and Tables

**Table 1:** Switch from or to ziprasidone: clinical data.

	ZIP+ switch to ziprasidone	ZIP− switch from ziprasidone	Difference
*N*	24	27	n.s.^b^
Diagnoses (ICD-10)			n.s.^b^
F20.0	18 (75.0%)	21 (77.8%)	
F20.1	2 (8.3%)	1 (3.7%)	
F20.3	4 (4.2%)	3 (11.1%)	
F20.4	0	2 (7.4%)	

Comorbidity (ICD-10)			
None	6 (25.0%)	16 (59.3%)	*P* = 0.025^b^
1	11 (45.8%)	9 (33.3%)	
2	7 (29.2%)	2 (7.4)%)	
F1 substance abuse^c^	12 (50.0%)	8 (29.6%)	n.s^b^
F43 adjustment disorder	6 (25.0%)	2 (7.4%)	*P* = 0.085^b^
F60 personality disorder	2 (8.3%)	1 (3.7%)	n.s.^b^

Women	16 (67%)	10 (37%)	*P* = 0.035^b^
Age	40.6 (8.9)	47.2 (10.3)	*P* = 0.017^a^
BMI at admission (kg/m²)	26.9 (3.2)	25.1 (2.7)	*P* = 0.042^a^
Women	26.9 (3.8)	24.0 (2.4)	*P* = 0.047^a^
Men	26.8 (1.5)	25.7 (2.8)	n.s^a^
Switch modalities			
Start/stop (abrupt change)	16 (67%)	17 (63%)	n.s.^b^
Tapering time (days)	2.3 (3.5)	2.4 (3.6)	n.s.^a^
Illness duration (years)	14.3 (7.8)	16.4 (10.0)	n.s.^a^
Hospitalization (days)	40.0 (21.2)	46.1 (43.7)	n.s.^a^

Ziprasidone dose	140.8 (27.8)	136.3 (38.0)	n.s.^a^
Minimum	80	80	
Maximum	180	240	
CPZE before switch	889 (291)	681 (190)	*P* = 0.004^a^
CPZE after switch	704 (140)	831 (340)	*P* = 0.094^a^

Psychotropic comedication: none	5 (20.8%)	5 (18.5%)	n.s.^b^

Laboratory abnormalities^d^	3 (12.5%)	1 (3.7%)	n.s.^b^
ECG abnormalities^e^	4 (16.7%)	4 (14.8%)	n.s.^b^
QTc prolongation >460 msec	0	0	n.s.^b^

Severity at admission	5.3 (0.61)	5.2 (0.64)	n.s.^a^
Severity at discharge	3.2 (0.89)	3.0 (0.85)	n.s.^a^
Change severity	2.0 (0.95)	2.2 (1.08)	n.s.^a^

Side effects at admission	1.1 (0.88)	0.9 (0.70)	n.s.^a^
Side effects at discharge	0.5 (0.59)	0.6 (0.64)	n.s.^a^
Change side effects	0.5 (0.78)	0.3 (0.62)	n.s.^a^

^a^ *t*-test (independent groups); ^b^ *χ* ^2^ test; ^c^F1 including alcohol abuse/dependence, THC abuse/dependence, benzodiazepine dependence, opioid abuse, multiple substance abuse (excluded: nicotine dependence); ^d^ZIP+: creatine kinase (2-fold), hyperglycemia, yGT (189 U/L); ZIP−: serum creatinine (1.4 mg/dL); ^e^ZIP+: AVB I°, repolarisation disturbance, nonspecific conduction disturbance (2); ZIP−: AVB I° (2), nonspecific conduction disturbance, abnormal repolarisation; n.s.: not significant; severity of illness was judged by 7-point ratings (CGI), side effects by a global severity rating (0–3).

**Table 2:** Switching from or to ZIP: psychotropic medication.

	ZIP+ switch to ziprasidone	ZIP− switch from ziprasidone
Antipsychotic medication before	Amisulpride (1)	Amisulpride (2)
switch/after switch^a^	Aripiprazole (2)	Aripiprazole (2)
		Clozapine (3)
	Flupenthixol (4)	
	Haloperidol (2)	
	Olanzapine (2)	Olanzapine (1)
		Paliperidone (4)
	Perazine (1)	Perazine (1)
	Quetiapine (5)	Quetiapine (7)
	Risperidone (7)	Risperidone (6)
		None (1)

Comedication at discharge^b^		
Low-potency neuroleptics	Chlorprothixene (1)	Chlorprothixene (4)
	Promethazine (1)	Promethazine (1)
	Melperone (6)	Melperone (4)
	Pipamperone (3)	Pipamperone (3)
Mood stabilizer	Valproic acid (3)	Valproic acid (1)
	Pregabalin (2)	Carbamazepine (1)
Antidepressants		Doxepin (1)
	Mirtazapine (2)	Mirtazapine (2)
	Citalopram (3)	
	Sertraline (1)	Sertraline (1)
	Trimipramine (1)	
	Moclobemide (1)	
Other	Benzodiazepine (8)	Benzodiazepine (10)
	Biperiden (1)	Biperiden (3)

^a^monotherapy; ^b^combination treatment in 57% of patients (29/51).

**Table 3:** Switching to or from ziprasidone-reasons for switch of antipsychotics.

Reason for switch	ZIP+ switch to ziprasidone	ZIP− switch from ziprasidone	Difference *χ* ^2^ test
*N*	24	27	
Positive symptoms^a^	6 (25.0%)	13 (48.1%)	*P* = 0.088
Depression^a^	6 (25.0%)	2 (7.4%)	*P* = 0.085
Negative symptoms^a^	5 (20.8%)	7 (25.9%)	n.s.
Sedation^b^	1 (4.2%)	3 (11.1%)	n.s.
Weight gain^b^	8 (33.3%)	1 (3.7%)	*P* = 0.006
Agitation^b^	—	1 (3.7%)	n.s.
EPS^b^	5 (20.8%)	1 (3.7%)	*P* = 0.058
Other side effects^b^	2 (8.3%)	1 (3.7%)	n.s.
Patient's choice^c^	4 (16.7%)	10 (37.0%)	*P* = 0.10

More than one reason possible (multiple entries); ^a^lack of effectiveness; ^b^side effects; ^c^patients' choice; EPS, extrapyramidal symptoms.
